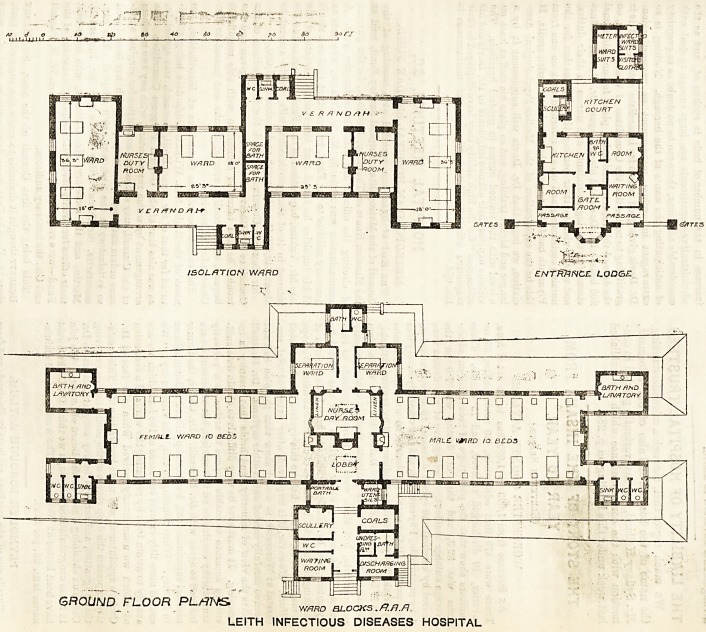# Hospital Construction

**Published:** 1899-01-28

**Authors:** 


### THE INSTITUTIONAL WORKSHOP.

### HOSPITAL CONSTRUCTION.

### LEITH PUBLIC HEALTH HOSPITAL.

THE name of this institution, as given above, is a pleasing euphemism for an infectious diseases hospital, which it really is, and expresses accurately enough the fact that the sole object of its erection is the public good. Designed by the late Mr. James Simpson, and completed under his son, Mr. George Simpson, it was opened two years ago, and has cost about £46,000, or £390 per bed for the 120 patients accommodated, while the site of about seven acres, two miles from the Forth, has cost £4,000 more. No doubt the cost of land has influenced the general plan, for we find the main pavilions are less than 100 ft. apart, and their nearest portions are only 35 ft. from each other. Taking the administration block first, it should be noted that it is in itself somewhat cramped, for the main corridor is lighted only by skylights, and the doctors' lavatory and bath-room is lighted and ventilated only through a w.c., which itself appears to open only below the skylights

A A A, Ward Blocks. B, Isolation Block. C, Administration Block. D, Entrance Lodge. E, Mortuary. F, Stables and Ambulance. G, Coachman's House. H, Blanket, Linen, and Clothes Store. J, Disinfector. K, Laundry, Engine-room, Workshops, and Disincharging Block. fector.

LEITH INFECTIOUS DISEASES HOSPITAL.

of the above mentioned corridor. Again, in the attached offices, we find the servants' bath and w.c. contained in one small room, a defect which could surely have been avoided at a nominal expense. The entrance lodge contains the same feature, but is otherwise suitably arranged. The laundry block contains the engine and boiler rooms, disinfectors and destructors, the latter

GROUND FLOOR PLANS. WARD BLOCKS. A.A.A.

LEITH INFECTIOUS DISEASES HOSPITAL.

being combined with the boiler furnaces. The patients are accommodated in four double pavilions and an isolation block, the latter containing 10 beds, the former 22 beds each, and all being only one storey high. The w.c.'s and baths are in separate annexes at the end of each main ward, with cross ventilated passages, and there is a separate annexe, with bath and w.c., for the two separation wards. The nurses' day (or duty) room and the entrance lobby are lighted only from the top, and must suffer in ventilation, while the absence of external windows from the former is a distinct disadvantage. The warming is by hot-water coils supplied with fresh air, placed between each bed, but there are fire-places as well, with ventilating grates, placed at each end of the main wards. There are windows between the last bed and the end of the ward, but none in the end wall itself, and these might have been introduced with advantage where the end is to the south. The isolation block is really two small hospitals for five beds each, and is constructed in accordance with the suggestions of the model plan of the Local Government Board. The drains discharge into the sea, and it does not appear that the sewage is subjected to previous treatment. The lighting is by gas, and there is complete telephonic communication between the departments. The plan, speaking generally, has evidently been carefully considered, with satisfactory results, but economy would seem to have been unduly studied in some of the arrangements adverted to above.

## Figures and Tables

**Figure f1:**
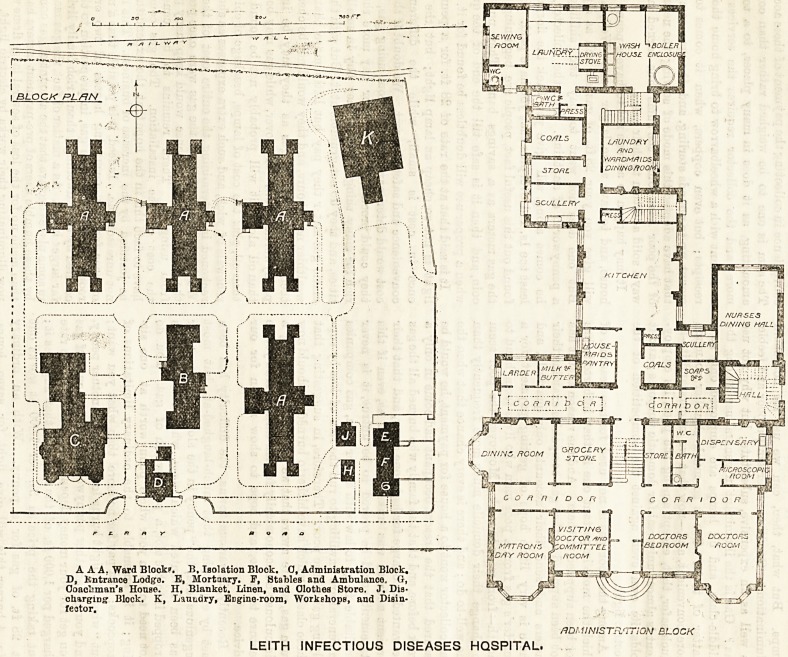


**Figure f2:**